# Single‐cell molecular profiling provides a high‐resolution map of basophil and mast cell development

**DOI:** 10.1111/all.14633

**Published:** 2020-11-05

**Authors:** Fiona K. Hamey, Winnie W.Y. Lau, Iwo Kucinski, Xiaonan Wang, Evangelia Diamanti, Nicola K. Wilson, Berthold Göttgens, Joakim S. Dahlin

**Affiliations:** ^1^ Department of Haematology Wellcome–Medical Research Council Cambridge Stem Cell Institute University of Cambridge Cambridge UK; ^2^ Present address: JDRF/Wellcome Diabetes and Inflammation Laboratory Wellcome Centre for Human Genetics University of Oxford Oxford UK; ^3^ Department of Medicine Karolinska Institutet and Karolinska University Hospital Stockholm Sweden

**Keywords:** basophils, differentiation, mast cells, single‐cell RNA sequencing, transcriptomics

## Abstract

**Background:**

Basophils and mast cells contribute to the development of allergic reactions. Whereas these mature effector cells are extensively studied, the differentiation trajectories from hematopoietic progenitors to basophils and mast cells are largely uncharted at the single‐cell level.

**Methods:**

We performed multicolor flow cytometry, high‐coverage single‐cell RNA sequencing analyses, and cell fate assays to chart basophil and mast cell differentiation at single‐cell resolution in mouse.

**Results:**

Analysis of flow cytometry data reconstructed a detailed map of basophil and mast cell differentiation, including a bifurcation of progenitors into two specific trajectories. Molecular profiling and pseudotime ordering of the single cells revealed gene expression changes during differentiation. Cell fate assays showed that multicolor flow cytometry and transcriptional profiling successfully predict the bipotent phenotype of a previously uncharacterized population of peritoneal basophil‐mast cell progenitors.

**Conclusions:**

A combination of molecular and functional profiling of bone marrow and peritoneal cells provided a detailed road map of basophil and mast cell development. An interactive web resource was created to enable the wider research community to explore the expression dynamics for any gene of interest.

## Graphical abstract

Flow cytometry and single‐cell gene expression data reconstruct a road map of mouse basophil and mast cell differentiation. Cell fate assays show that previously uncharacterized peritoneal progenitors can differentiate into both basophils and mast cells. An interactive web resource enables the wider research community to explore the gene expression dynamics of differentiating cells.

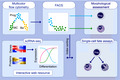

Abbreviations: Ba, basophil; FACS, fluorescence‐activated cell sorting; MC, mast cell; Prog, progenitor; scRNA‐seq, single‐cell RNA sequencing.

## 1 INTRODUCTION

Mast cells are sentinel cells that are strategically positioned throughout the body and allow rapid triggering of the immune system upon infections.^1^ Mast cell activation also follows IgE‐allergen‐mediated crosslinking of the FcεRI receptors in atopic individuals, which causes an allergic reaction. Along with basophils, activation of mast cells results in prompt release of proteases and histamine from the cytoplasmic granules as well as synthesis of cytokines and chemokines. These mediators in turn cause inflammation, vasodilation, and leukocyte recruitment to the site of triggering.^1^ Thus, the functions of mature basophils and mast cells have been studied in great detail. However, less is known about these cells’ development.

A hierarchical model with distinct megakaryocyte‐erythroid, granulocyte‐monocyte, and lymphoid branches, was until recently the dominating representation of hematopoiesis.^2^ Single‐cell RNA sequencing (scRNA‐seq) coupled with cell fate assays now reveals that hematopoietic differentiation more likely represents a landscape of cell states with continuous progression from multi‐ and bipotent progenitors into each respective cell lineage.^3^, ^4^, ^5^, ^6^, ^7^ In particular, single‐cell transcriptomics of Lin^−^ c‐Kit^+^ mouse bone marrow progenitors uncovers a continuous differentiation from hematopoietic stem cells to bipotent basophil‐mast cell progenitors (BMCPs).^4^ Microarray analysis of bulk‐sorted cells shows distinct gene expression profiles of mature basophils and mast cells.^8^ However, investigation of temporal gene expression dynamics during basophil and mast cell specification and maturation is yet to be delineated and requires single‐cell resolution.

Here, we combine multicolor flow cytometry‐based index sorting with high‐coverage scRNA‐seq to investigate the basophil‐mast cell bifurcation and the differentiation into each respective lineage. We demonstrate that molecular profiling and pseudotime ordering of single cells highlights genes that are critical for cell differentiation and maturation. The analysis is accompanied with the generation of a user‐friendly web resource that allows gene expression to be explored across the single‐cell landscape. Finally, we use cell fate assays to show that single‐cell transcriptomics and protein epitope data analysis successfully predict the fate potential of the previously uncharacterized BMCP population in the peritoneal cavity. Taken together, the current resource provides a detailed road map of the developmentally related basophils and mast cells, whose activation contributes to allergic diseases.

## 2 METHODS

### 2.1 Cell isolation and flow cytometry

Experiments involving mice were performed according to the United Kingdom Home Office regulations. PBS with 2% fetal calf serum (Sigma‐Aldrich, St Louis, MO) and 1 mmol/L EDTA was injected into the peritoneal cavity of euthanized C57BL/6 mice. The fluid was aspirated following vigorous massage, and the cells were prepared for FACS. Peritoneal lavage samples with excessive blood contamination were discarded before data acquisition. Bone marrow cells were extracted by flushing or crushing the femurs, tibias, and/or ilia. Red blood cells were lysed, and the remaining cells were prepared for FACS. The cells were sorted with a BD Influx cell sorter (BD Biosciences, San Jose, CA). Cell doublets were excluded with the width parameters. P1 cells and mast cells were sorted two consecutive times for cell culture experiments. The cells were sorted into Terasaki plates (Greiner Bio‐One, Kremsmünster, Austria) or 96‐well plate wells. Visual inspection determined colony sizes following culture, and the size was set to 1 if no live cells were observed in a particular well. Flow cytometry was typically performed on colonies constituting at least 20 cells, and potential to form a particular cell lineage was based on at least 5 events in a given gate, as described previously.^4^ Cultured cells were analyzed with the BD Fortessa flow cytometers (BD Biosciences).

### 2.2 Antibodies and cell staining

Primary cells were incubated with the antibodies integrin β7 (clone FIB504), CD34 (RAM34), Sca‐1 (D7), CD16/32 (93), c‐Kit (2B8), FcεRI (MAR‐1), IL‐33Rα/ST2 (DIH9), and/or CD49b (DX5). The Easysep mouse hematopoietic progenitor cell isolation cocktail (STEMCELL Technologies, Vancouver, Canada) stained lineage markers. Cultured cells were stained with c‐Kit, FcεRI, CD49b, with or without TER119 (TER119). Fc‐block (clone 93) was used where appropriate. The antibodies were from BD Biosciences, Biolegend (San Diego, CA), and Thermo Fisher Scientific (Waltham, MA). DAPI (BD Biosciences) or 7‐AAD (Thermo Fisher Scientific) were used to exclude dead cells.

### 2.3 Cell culture

The cells were cultured for 6‐7 days in IMDM (Sigma‐Aldrich) with 20% heat‐inactivated fetal calf serum (Sigma‐Aldrich), 100 U/ml penicillin (Sigma‐Aldrich), 0.1 mg/mL streptomycin (Sigma‐Aldrich), and 50‐200 μmol/L β‐mercaptoethanol (Thermo Fisher Scientific). The medium was supplemented with 20 ng/mL IL‐3 and 100 ng/mL stem cell factor, or 80 ng/mL stem cell factor, 20 ng/mL IL‐3, 50 ng/mL IL‐9, and 2 U/mL erythropoietin. All cytokines were recombinant mouse cytokines (Peprotech, Rocky Hill, NJ) except the erythropoietin (Eprex; Janssen‐Cilag, High Wycombe, UK), which was human.

### 2.4 Flow cytometry analysis

FlowJo v10 (Treestar, Ashland, OR) produced the flow cytometry plots. Diffusion map and principal component analysis (PCA) plots of flow cytometry data were generated using the R programming environment. The flow cytometry events were down‐sampled according to the population with the least number of events. Duplicate entries were removed, and the parameters representing fluorescent markers log‐transformed. Variables were *z*‐scored and diffusion map plots generated using the *destiny* and *ggplot2* packages. PCA was calculated using the prcomp function. Data projection was performed using the predict function.

### 2.5 scRNA‐seq data analysis

Primary single cells were FACS index sorted into lysis buffer, and scRNA‐seq was performed based on the Smart‐Seq2 protocol.^9^ For details of scRNA‐seq data processing, see Supplementary methods. Analysis was performed using the scanpy v1.4 python module^10^ and the R programming environment. Interactive websites for plotting gene expression and flow cytometry data are hosted at http://128.232.224.252/bas/ and http://128.232.224.252/per/ for the basophil and mast cell dataset, respectively.

### 2.6 Data sharing statement

Protocols and scRNA‐seq data generated for this article have been deposited in the Gene Expression Omnibus database (accession numbers GSE128003 and GSE128074). scRNA‐seq data of bone marrow BMCPs, analyzed in Dahlin et al,^4^ are available through GSE106973. Human scRNA‐seq data were obtained from the Human Cell Atlas.^11^ For other original data, please contact joakim.dahlin@ki.se or bg200@cam.ac.uk.

## 3 RESULTS

### 3.1 Multicolor flow cytometry analysis reveals the basophil and mast cell differentiation trajectories

**Figure 1:**
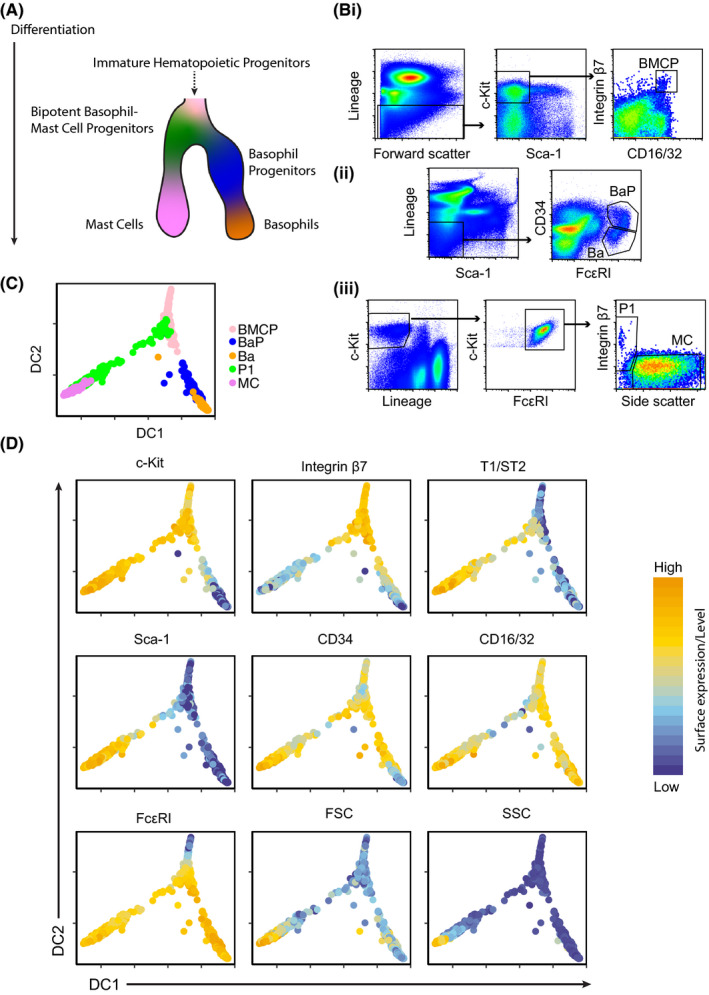
Flow cytometry analysis reveals differentiation trajectories from bipotent basophil‐mast cell progenitors to basophils and mast cells. A, Illustration outlining the basophil and mast cell differentiation trajectories. B, Flow cytometry‐based gating strategies of (Bi) bipotent basophil‐mast cell progenitors (BMCPs) from bone marrow, (Bii) basophil progenitors (BaP) and basophils (Ba) from bone marrow, and (Biii) P1 cells and mast cells from peritoneal cavity. Lineage markers include 7‐4, CD5, CD11b, CD19, CD45R/B220, Ly6G/C (Gr‐1), and TER119. C, Diffusion map visualization of the flow cytometry data colored by cell type. D, Diffusion map visualization of the flow cytometry data colored by protein expression or light scatter parameters. The surface expression parameters and light scatter parameters are visualized on log‐transformed and linear scales, respectively. Expression of lineage markers and viability staining are not shown. The data are representative of 4 independent experiments

Basophil and mast cell differentiation are closely linked, and the cells share a common bipotent progenitor (Figure 1A). Here, we used multicolor flow cytometry to map these branching trajectories at the single‐cell level. Flow cytometry analysis of mouse bone marrow cells captured BMCPs and cells of the basophil differentiation trajectory (Figure 1Bi,ii).^4^, ^12^ We performed parallel analysis of Lin^‐^ c‐Kit^+^ FcεRI^+^ peritoneal cells in an attempt to capture late mast cell differentiation, which takes place at peripheral sites. Analysis of the Lin^‐^ c‐Kit^+^ FcεRI^+^ peritoneal cells distinguished two populations based on integrin β7 expression and cell granularity (measured with the side scatter parameter). A broad gate that included a continuum of prospective mast cell progenitors, intermediate precursors, and mast cells—hereon referred to as population P1—was set in close proximity to the mast cell gate (Figure 1Biii). To enable 2‐dimensional visualization of the flow cytometry single‐cell datasets, we performed dimensionality reduction using a diffusion map algorithm.^13^ This method embeds a dataset by considering the properties of random walks between cells that are close together in the high‐dimensional space, and can visualize branching cell differentiation trajectories in single‐cell data.^14^ Flow cytometry data covered the 5 cell populations recorded with 9 fluorescent and 2 light scatter parameters. The diffusion map revealed a bifurcation at the BMCP stage, establishing the putative entry points to the basophil and mast cell trajectories (Figure 1C). The diffusion map embedding further visualized the progression from BMCP, through basophil progenitors, to basophils. The mast cell trajectory exhibited a similar pattern, with differentiation of BMCPs to mature mast cells.

Plotting individual surface markers in the diffusion map allowed us to investigate how the proteins are expressed during differentiation. For example, loss of CD34 in combination with downregulation of c‐Kit marked the progression from BMCPs to basophils (Figure 1D), and loss of integrin β7 in c‐Kit^+^ cells was associated with differentiation along the trajectory from BMCPs to mast cells (Figure 1D). Taken together, the flow cytometry dataset provides a template of basophil‐mast cell differentiation at single‐cell level and highlights the bifurcation toward the two lineages.

### 3.2 Single‐cell profiling captures progression of basophil differentiation in the bone marrow

**Figure 2:**
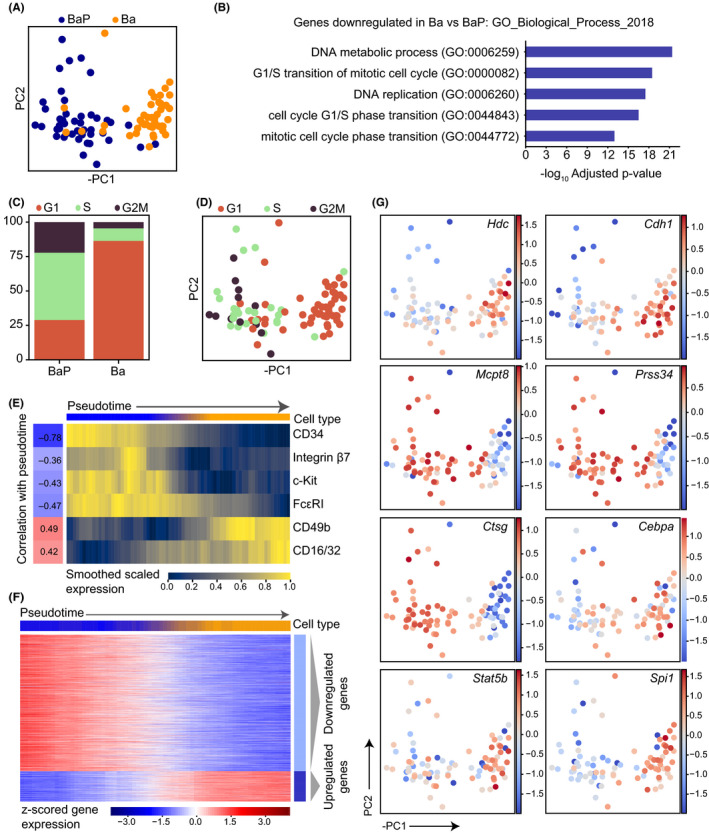
Bone marrow basophil progenitors downregulate cell cycle genes during differentiation. A, PCA of scRNA‐seq profiles colored by cell surface marker phenotype. PC, principal component. B, Top 5 GO Biological Process terms associated with the genes significantly upregulated in BaP cells compared to Ba cells, ranked by adjusted *P*‐value. Benjamini‐Hochberg correction for multiple hypotheses testing. Genes upregulated in Ba compared to BaP are presented in Figure S1B. C, Proportion of scRNA‐seq profiles from each phenotype computationally assigned to G1, S or G2M cell cycle states based on gene expression using the scanpy *score_genes_cell_cycle* function. D, PCA colored by cell cycle state. E, Levels of cell surface markers for cells ordered by PC1 pseudotime. Index data values were log‐transformed, smoothed along pseudotime by using a sliding window of size 20 and scaled between 0 and 1 for each marker. Correlation values indicate the pearson correlation coefficient between pseudotime and the unsmoothed expression values for each surface marker. Colorbar at the top indicates the phenotypic cell type proportions within each window. Blue corresponds to entirely BaPs and orange to Ba cells. F, Heatmap displaying the expression of genes dynamically expressed along the PC1 pseudotime ordering. The top colorbar indicates the cell type proportion in each window. Expression is smoothed along a sliding window and *z*‐scored for each gene, and genes were clustered using Louvain clustering into groups showing different dynamics. Dynamic genes defined as adjusted *P*‐value < .01 in permutation test, details in supplementary methods. G, PCA colored by *z*‐scored expression of specific genes. The data represent cells pooled from 3 individual mice

Analysis by flow cytometry suggested that the flow cytometry gating strategies we used could be capturing a continuum of differentiation toward basophils and mast cells. To first identify changes in gene expression programs during basophil differentiation, we performed scRNA‐seq of primary basophil progenitor (BaP) cells and basophil (Ba) cells from mouse bone marrow. Both PCA and diffusion maps showed separation between the majority of cells from the two sorting gates (Figure 2A, Figure S1A). To investigate which genes were driving this separation, we performed differential expression analysis, identifying 212 upregulated and 833 downregulated genes in Ba cells compared to BaPs (adjusted *P*‐value < .01, t test with Benjamini‐Hochberg correction) (Table S1). Enrichment analysis of these gene lists revealed that upregulated genes were enriched for granulocyte immune response terms (Table S2, Figure S1B). Downregulated genes were enriched for cell cycle related terms (Table S2, Figure 2B), suggesting a difference in cell cycle behavior throughout the differentiation process. This observation is in line with other hematopoietic differentiation pathways, where progenitors commonly loose proliferative capacity as they mature into the fully differentiated cell types.

To further explore this, we then performed analysis to computationally assign cell cycle state to the single‐cell profiles.^15^ Consistent with the gene list enrichment analysis, the majority of cells in the BaP gate were assigned to S and G2M states (69%), whereas 87% of cells in the Ba gate were assigned to G1 state (Figure 2C, D). The effect of cell cycle status was clear in the diffusion map dimensionality reduction (Figure S1C), confounding attempts to order cells using pseudotime algorithms. Instead, downregulation of progenitor marker genes such as *Cd34* and *Kit* indicated that ordering cells along PC1 could be used to arrange cells in pseudotime (Figure S1D). Visualization of index sorting data also showed clear dynamics of the different surface markers along PC1 (Figure 2E). As expected, CD34 and c‐Kit protein expression showed a negative correlation with pseudotime (compare Figures 1D and 2E), which indicates their downregulation during basophil differentiation. In addition, the basophil marker CD49b (DX5) showed a positive correlation with pseudotime ordering (Figure 2E).

Using the PC1 pseudotime ordering, we then identified genes that dynamically changed during differentiation (Figure 2F). Clustering sorted these dynamic genes into two groups: one increasing and one decreasing with differentiation (Table S3). Basophil differentiation was associated with upregulation of *Hdc*, which is associated with histamine synthesis, and increased expression of the basophil gene E‐cadherin (*Cdh1*). We further observed downregulation of the proteases *Mcpt8*, *Prss34,* and *Ctsg* and upregulation of the transcription factors *Cebpa*, *Stat5b*, and *Spi1* (Figure 2G). To validate the full lists of dynamically regulated genes, we compared these to mast cell and basophil signature genes identified using bulk microarray analysis.^8^ Genes upregulated during basophil differentiation exhibited a significant overlap with the previously described basophil signature genes (*P* = 4.0 × 10^−29^, hypergeometric test, Figure S1Ei), whereas genes that were downregulated during differentiation had significant overlap with the previously described mast cell signature genes (*P* = 1.3 × 10^−4^, hypergeometric test, Figure S1Eii).

Together, this analysis offers a description of the dynamics of gene expression during basophil differentiation and highlights changes in cell cycle activity as one of the major occurrences during this maturation process.

### 3.3 Single‐cell gene expression analysis suggests a continuum of mast cell differentiation in the peritoneal cavity

**Figure 3:**
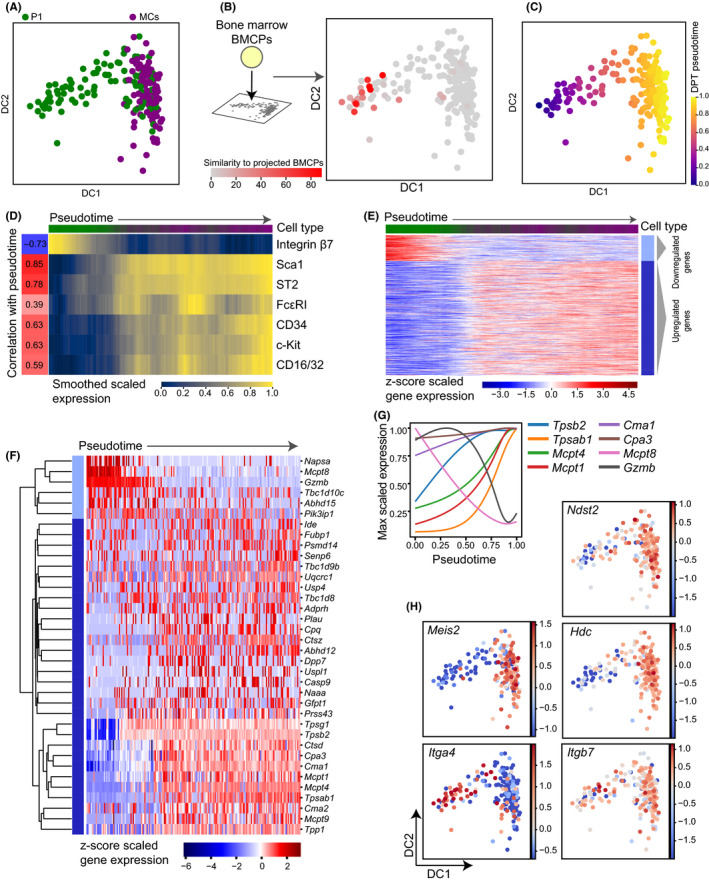
Transcriptional profiling of peritoneal mast cell progenitors captures a differentiation continuum. A, Diffusion map dimensionality reduction of scRNA‐seq profiles colored by cell phenotype. DC, diffusion component. B, Bone marrow BMCP cells from Dahlin et al^4^ were projected into the PCA space of the peritoneal cells and the 10 closest peritoneal neighbors of each bone marrow cell were identified in these co‐ordinates. Cells are colored by a score representing how frequently each peritoneal cell was the nearest neighbor of a bone marrow BMCP. C, Diffusion map colored by pseudotime ordering of cells. DPT, diffusion pseudotime. D, Levels of cell surface markers for pseudotime ordered cells. Index data values were log‐transformed, smoothed along pseudotime by using a sliding window of size 20 and scaled between 0 and 1 for each marker. Correlation values indicate the pearson correlation coefficient between pseudotime and the unsmoothed expression values for each surface marker. Colorbar at the top indicates the phenotypic cell type proportions within each window. Green corresponds to entirely P1 cells and purple to MCs. E, Heatmap displaying the expression of genes dynamically expressed along the pseudotime ordering. The top colorbar indicates the proportion of cell type in each window. Expression is smoothed along a sliding window and z‐scored for each gene, and genes were clustered using Louvain clustering into groups showing different dynamics. Dynamic genes defined as adjusted *P*‐value < .01 in permutation test, details in supplementary methods. F, Heatmap of dynamically regulated proteases showing *z*‐scored gene expression along pseudotime. Genes were ordered using the hierarchical clustering indicated by the dendrogram. Colorbar indicates the Louvain cluster from (E) for each gene. G, Expression trends of specific genes along pseudotime. Genes are scaled by their maximum expression value rather than z‐scoring as in the heatmap. H, Diffusion map colored by z‐score scaled expression of specific genes. The data represent cells pooled from 4 individual mice

After exploring the basophil progenitors, we next decided to focus on mast cell differentiation in the peritoneal cavity. The flow cytometry data suggested the existence of both peritoneal BMCPs and mast cells (Figure 1), so we performed single‐cell RNA sequencing on these primary cell populations to characterize them based on gene expression. A subset of the P1 cells clustered separately from the mast cells in the diffusion map plot, demonstrating a difference between the transcriptome of these cells and the peritoneal mast cells (Figure 3A). In previous work, we characterized bone marrow BMCPs at the single‐cell gene expression level.^4^ To examine the similarity of these bone marrow progenitors to the peritoneal mast cell differentiation, single‐cell bone marrow BMCP profiles from Dahlin et al^4^ were projected onto the peritoneal dataset (Figure 3B). This demonstrated that the P1 peritoneal cells furthest from the peritoneal MCs were most similar to the bone marrow BMCPs, supporting that these were the most immature cells in the dataset.

To understand expression changes during mast cell maturation, we then performed pseudotime ordering of the peritoneal cells (Figure 3C). As expected, interrogation of cell surface markers along pseudotime showed a strong downregulation of integrin β7 and strong upregulation of markers such as Sca1 and ST2 (compare Figures 1D and 3D). Genes exhibiting dynamic expression patterns were identified and clustered as for the basophil trajectory (Table S4, Figure 3E). Annotation from the Panther database^16^ was used to interrogate the two gene clusters for overlap with specific annotated gene sets such as proteases. Protease genes downregulated during mast cell differentiation included *Mcpt8* and *Gzmb*, whereas *Cpa3*, *Cma1*, *Mcpt1*, *Mcpt4*, *Tpsb2,* and *Tpsab1* increased with differentiation (Figure 3F). To investigate the temporal induction and loss of protease genes, we changed visualization method and scaled the gene expression according to the cell with maximum expression (instead of z‐scoring genes across the dataset). Early‐onset proteases included *Cpa3*, followed by *Tpsb2*, and finally *Tpsab1*, indicating that the protease induction occurs in stages (Figure 3G, raw values for individual genes shown in Figure S2C).

To validate the full lists of dynamically regulated genes in the peritoneal mast cell dataset, we compared these to mast cell and basophil signatures identified in Dwyer et al.^8^ The upregulated genes significantly overlapped with the mast cell signature genes (*P* = 3.7 × 10^−65^, hypergeometric test, Figure S2Di), including *Ndst2* and *Meis2* (Figure 3H). Some genes showed expression enrichment mainly in the mast cells (*Meis2*), whereas others were expressed more evenly across the trajectory save for lower expression at the beginning of pseudotime (*Ndst2*). Similar to basophil differentiation, mast cell differentiation was associated with *Hdc* upregulation (Figure 3H). There was also a small overlap between the downregulated genes and basophil signature genes (*P* = 2.5 × 10^−5^, hypergeometric test, Figure S2Dii). To investigate the link between gene and protein expression, we also interrogated the expression of *Itga4* and *Itgb7*, which encode subunits of integrin β7. *Itga4* was significantly downregulated with a similar expression pattern to integrin β7 in the flow cytometry data whereas *Itgb7* was not significantly changing in pseudotime (Figure 3D, H).

### 3.4 P1 cells in the peritoneal cavity exhibit basophil and mast cell‐forming potential

**Figure 4:**
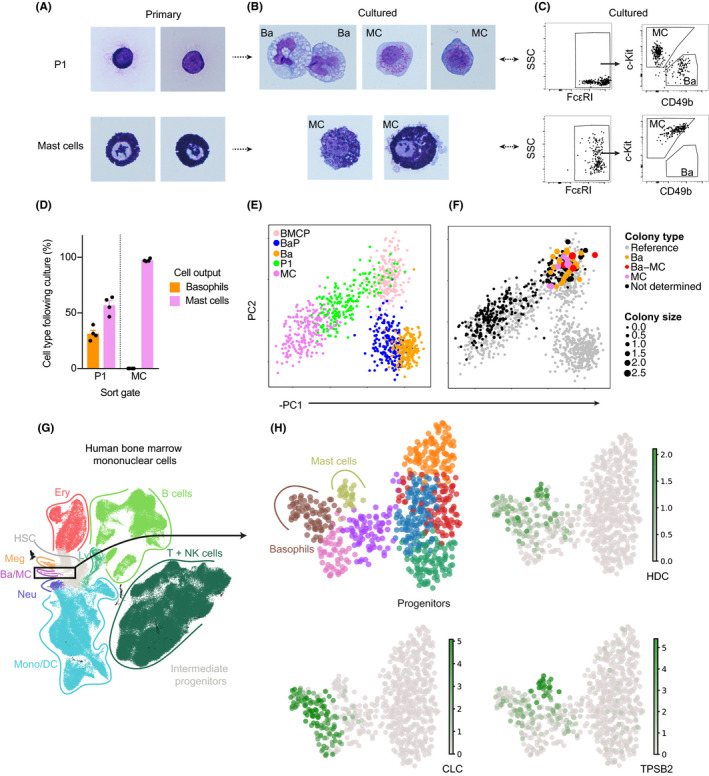
P1 peritoneal cells exhibit potential to form basophils and mast cells. A‐B, May‐Grünwald Giemsa staining of primary and in vitro cultured P1 cells and mast cells extracted from the peritoneal cavity. Ba, basophil; MC, mast cell. Two or seven independent experiments revealed the morphology of primary P1 cells and mast cells, respectively. C, Flow cytometry gating strategy to identify basophils and mast cells cultured from primary P1 cells and mast cells. D, Quantification of cell type output following bulk‐culture and flow cytometry analysis of P1 cells and mast cells. Pooled data from 4 independent experiments per population are shown. The means and SEMs are shown. E, Principal component analysis of the flow cytometry reference dataset, provided in Figure 1C, colored by cell type. F, Projection of index‐sorted cells into the principal component space of the reference dataset. The point size represents log_10_‐transformed colony size and the colors represent colony type following cell culture. Panel F shows data pooled from 2 independent experiments. The cells were cultured with IL‐3 and stem cell factor. G, UMAP visualization of the Human Cell Atlas single‐cell transcriptomics data colored by cell type. Identity of the clusters was assigned based on established marker genes. H, UMAP visualization of the basophil‐mast cell differentiation trajectory colored by cluster or expression of different genes

The flow cytometry‐based and transcriptional analyses revealed an immature cell population with BMCP‐like characteristics in the peritoneal cavity. However, a population of bipotent peritoneal BMCPs has not previously been described at this site. We therefore explored whether the protein and transcriptional analyses successfully predicted the developmental state of the peritoneal P1 cells and mast cells. Fluorescence‐activated cell sorting (FACS) isolated P1 cells and mast cells were cytochemically stained with May‐Grünwald Giemsa. Primary P1 cells displayed little cytoplasm that contained no or few granules, consistent with the morphology of blasts (Figure 4A). In contrast, primary mast cells were filled with numerous metachromatic granules, in agreement with a mature morphology (Figure 4A).

We cultured the peritoneal cells to investigate whether the P1 cell population exhibited capacity to generate basophils and mast cells. P1 cells cultured with IL‐3 and stem cell factor generated c‐Kit^‐^ FcεRI^+^ CD49b^+^ basophils and c‐Kit^+^ FcεRI^+^ mast cells, whereas primary mast cells only displayed mast cell‐forming capacity (Figure 4B‐D).

By contrast to bulk cultured cells, only cell fate assays performed at the single‐cell level have the potential to reveal whether the P1 population consists of bipotent progenitors. Therefore, single P1 cells and mast cells were index sorted into individual wells, the resulting colony sizes were measured, and the colonies were subjected to flow cytometry analysis and cytochemical staining. To visualize the cell culture data, we first performed PCA of the flow cytometry data presented in Figure 1C, henceforth referred to as the reference dataset (Figure 4E). We then projected the FACS index sort data onto the principal component space of the reference dataset, and plotted colony size and colony type data (Figure 4F). Analysis of colony sizes showed that colonies derived from P1 cells were large, whereas cells along the mast cell trajectory exhibited reduced proliferation rate (Figure 4F, Figure S3). Notably, the cell fate assays revealed that primary P1 cells formed pure basophil colonies, pure mast cell colonies or mixed basophil‐mast cell colonies (Figure 4F, Figure S3A). Colonies derived from single mast cells were too small to analyze with flow cytometry. However, mast cells cultured in bulk remained mast cells as expected (Figure 4C‐D, Figure S3B). Further analysis of the FACS index sort data revealed that primary cells that formed large colonies comprising basophils and/or mast cells were mainly integrin β7^+/hi^ P1 progenitors (Figure S4). This observation agrees with the pseudotime ordering of the single‐cell transcriptomics data, which showed that loss of integrin β7 is associated with differentiation. We also cultured the P1 peritoneal cells in erythroid‐promoting conditions, as the early basophil‐mast cell differentiation is closely linked to the erythrocyte trajectory.^3^ However, no erythroid output was observed (Figure S5), indicating that the P1 cells indeed consisted of bipotent basophil‐mast cell progenitors.

After investigating the bifurcation of bipotent BMCPs in mouse, we decided to explore single‐cell transcriptomics data in human to see whether it supports a similar relationship between basophil and mast cell differentiation. We processed data of human bone marrow cells from the Human Cell Atlas.^11^, ^17^ By subsetting the data for pertinent progenitor populations, we identified a distinct differentiation trajectory with a gene expression profile characteristic of basophils and mast cells (*HDC* and *MS4A2*), which was separate from other myelo‐erythroid lineages (Figure 4G‐H, Figure S6). Observed expression of basophil (*CLC* and *CEBPA*) and mast cell (*TPSB2* and *TPSAB1*) genes highlighted the gradual differentiation and entry points of the respective lineages (Figure 4H, Figure S6C). The observation of neighboring entry points indicated a close developmental relationship between human basophils and mast cells, whereas erythrocyte development progressed on a separate trajectory.

Taken together, the cell culture assays revealed that the protein and gene expression analyses successfully predicted the differentiation state of the P1 cell population in the peritoneal cavity.

## 4 DISCUSSION

Single‐cell transcriptomics coupled with index sorting of thousands of bone marrow HSPCs has previously been used to chart erythrocyte and granulocyte‐monocyte differentiation.^18^, ^19^ BMCPs represent a minor fraction of the bone marrow HSPCs, and capturing the early basophil‐mast cell axis therefore requires analysis of tens of thousands of HSPCs.^4^ The early differentiation of progenitors with mast cell‐forming capacity occurs in the bone marrow.^20^ However, full mast cell differentiation and maturation takes place at peripheral sites,^20^ and we therefore specifically sorted Lin^‐^ c‐Kit^+^ FcεRI^+^ cells extracted from the peritoneal cavity to capture this process. Cell isolation from peritoneum does not require enzymatic digestion, thus minimizing external stimuli during cell processing. Basophil differentiation takes place in bone marrow, and we therefore analyzed basophils and their progenitors from this site.

The single‐cell transcriptomics data presented here capture a continuum of cells from peritoneal BMCPs to mast cells. Recent studies have explored whether bone marrow HSPCs constitute the primary source of peritoneal mast cells. Transferred bone marrow cells contribute little to the peritoneal mast cell numbers unless the local pool of mature mast cells are depleted first.^21^, ^22^ A known feedback mechanism, in which mast cells inhibit recruitment and differentiation of their progenitors, provides a likely explanation to these observations.^23^, ^24^ The peritoneal mast cells in adult mice emerge from definitive hematopoiesis.^21^, ^22^ However, this observation does not necessarily imply that bone marrow HSPCs are the main source of mast cells. Further studies exploring the relationship between bone marrow HSPCs and peritoneal mast cell differentiation are therefore needed. It will also be important to generate reference maps of mast cell differentiation at alternative compartments and in the prenatal setting, given the heterogeneity of the mast cell population.

We reveal the existence of a progenitor with dual basophil‐mast cell‐forming potential in the peritoneal cavity. BMCPs have previously been described in the mouse spleen and bone marrow,^4^, ^12^, ^25^ and the presence of a bipotent progenitor population indicates that there is a close association between the basophil and mast cell differentiation trajectories. One study has questioned the bipotent nature of splenic BMCPs,^26^ as only mast cell colonies were observed following culture. The failure to detect basophils in that study is yet to be explained.

Recent data suggest that the erythroid axis is coupled with the basophil and/or mast cell fates.^3^, ^5^, ^27^, ^28^, ^29^ However, we did not observe erythrocyte‐forming potential among P1 cells in the peritoneum. In agreement with this, BMCPs in the spleen and bone marrow are unable to generate erythrocytes,^4^, ^12^ altogether suggesting that loss of erythrocyte‐forming potential is an early event along the differentiation trajectory from hematopoietic stem cells to basophil and mast cells. Similarly, the human single‐cell transcriptional landscape presented here reveals a unique trajectory of cells diverging into basophils and mast cells, separate from the erythroid trajectory. This observation is in agreement with recent studies that demonstrate the presence of human progenitor populations that produce basophils and mast cells.^5^, ^27^ The low proliferation capacity of mast cells complicates culture‐based approaches to determine whether the cell populations harbor bipotent basophil‐mast cell progenitors.^30^ Previous culture experiments suggest that the human basophil and eosinophil differentiation trajectories are adjacent to each other.^31^, ^32^ Simultaneous existence of bipotent basophil‐mast cell progenitors and basophil‐eosinophil progenitors is in line with the landscape model of hematopoiesis.^20^ However, the exact association between the mast cell, basophil, and eosinophil fates in mouse and human is still to be deciphered.

Temporal ordering of the cells in the transcriptomic datasets allows exploration and verification of molecular processes in differentiating basophils and mast cells. We show that *Ndst2* (encoding *N*‐deacetylase/*N*‐sulphotransferase‐2) is upregulated during differentiation from BMCPs to mature mast cells, and this was also associated with the appearance of numerous densely stained granules. In agreement with these findings, dense May‐Grünwald Giemsa staining of the peritoneal mast cell granules requires sulphated heparin, which is dependent on *Ndst2* expression.^33^ Mast cells and basophils are major producers of histamine, which is quickly released upon cell activation.^34^ Here, we verified that the expression of the enzyme that catalyzes the histamine synthesis, histidine decarboxylase (*Hdc*), increased upon differentiation of both basophils and mast cells. Analysis of the single‐cell transcriptomics data can also give insights into more complex regulatory processes. For example, integrin β7 expression on progenitors with mast cell‐forming potential is important for cell migration into the lungs in a mouse model of allergic airway inflammation.^35^ Downregulation of integrin β7 is a hallmark of terminal mast cell differentiation.^36^ However, we did not observe downregulation of *Itgb7* gene expression during the transition from BMCPs to mast cells, despite downregulation of the surface protein. Integrins constitute αβ heterodimers when localized to the cell surface, and further investigation into the gene expression profile revealed decreased expression of *Itga4*, the binding partner of the integrin β7 subunit, upon differentiation. Thus, the loss of integrin α4 gene expression likely explains the downregulation of integrin β7 protein expression on the cell surface.

*Mcpt1* expression is typically associated with mucosal mast cells. Nevertheless, *Mcpt1* was upregulated during differentiation of serosal‐type peritoneal mast cells. However, the levels detected were several orders of magnitude lower than the levels of *Tpsb2*, *Cma1*, and *Mcpt4*.

During basophil differentiation, the transcription factors *Stat5b* and *Cebpa* are upregulated along the progression of pseudotime. The expression of C/EBPα is STAT5‐dependent, and both genes are required for basophil formation.^12^, ^25^ Dynamic expression of transcription factors with currently unknown functions in basophil and mast cell differentiation was also recognized. For example, *Spi1*, which encodes PU.1, is upregulated during late basophil differentiation. It is known to be involved in neutrophil granulocyte maturation,^37^, ^38^ but the role of PU.1 in basophil differentiation is yet to be delineated. During mast cell differentiation, we describe the increase of the transcription factor *Meis2*. Primary mast cells from human skin express this transcription factor,^39^ but the potential function during mast cell differentiation is yet to be described.

Microarray and RNA sequencing analyses reported previously provide detailed gene expression patterns of mature hematopoietic cell populations, including bulk‐sorted mature basophils and mast cells.^8^, ^40^ We observed that differentiation into basophils and mast cells involves activation of mutually exclusive lineage programs. However, a small subset of the previously reported signature genes is not unique to mature cells, but can also be observed in bipotent progenitors. For example, we show that *Mcpt8* expression is not restricted to basophils but is also expressed by BMCPs. Indirect evidence also supports the validity of this observation.^41^, ^42^ Transient *Mcpt8* expression at the BMCP stage in fact provides an explanation to a major conundrum in the field. Basophils, identified as *Mcpt8*‐expressing cells, have been reported to exhibit potential to transdifferentiate into mast cells.^43^ Our results show that a more likely scenario is that a subset of the previously reported *Mcpt8*‐expressing cells constitutes bipotent BMCPs that can give rise to mast cells.

In summary, here we have reported the generation of a high‐resolution single‐cell map of the BMCP bifurcation and mast cell and basophil differentiation. A user‐friendly interactive website has been created for the wider community to enable further exploration of the data.

## CONFLICT OF INTEREST

The authors declare that they have no competing interests.

## AUTHORSHIP CONTRIBUTIONS

JSD and WWYL performed experiments; ED mapped sequencing data; FKH, JSD, and XW analyzed single‐cell RNA sequencing data; JSD analyzed flow cytometry and cell culture experiments; NKW contributed to important discussions; IK created the web resource and analyzed the Human Cell Atlas data; BG and JSD supervised the study; BG secured funding; FKH and JSD drafted the manuscript; and all authors contributed to final version of the manuscript.

## Supporting information

Fig S1‐6

Table S1

Table S2

Table S3

Table S4

Supplementary Material
