# Supplementary material for: Single‐cell molecular profiling provides a high‐resolution map of basophil and mast cell development
Source: Allergy. 2020 Nov 5;76(6):1731–42. doi: 10.1111/all.14633 (PMC8246912; doi:10.1111/all.14633)
Supplement: Supplementary file 1 — Fig S1‐6 [file ALL-76-1731-s006.pdf]

Supplementary Figure legends

Figure S1

(A) Diffusion map of Ba and BaP cells colored by their phenotypic cell type. DC, diffusion component. (B) Top 5 GO Biological Process terms associated with the genes significantly upregulated in Ba cells compared to BaP cells, ranked by adjusted p-value. Benjamini-Hochberg correction for multiple hypotheses testing. (C) Diffusion map colored by computationally assigned cell cycle state. (D) Diffusion map colored by expression of specific genes. (E) Overlap of basophil (Ba) differentiation up- (i) or down- (ii) regulated genes with mast cell and basophil signature gene sets from Dwyer et al. Significance of overlap was tested using a hypergeometric test, with resulting p-values displayed in figure. The Venn diagrams show genes annotated in Ensembl genome build 81 and Dwyer et al.

Figure S2

(A) PCA of peritoneal single-cell RNA-seq profiles showing outlier cells in PC1. PC, principal component. (B) Diffusion map dimensionality reduction colored by computationally assigned cell cycle state. DC, diffusion component. (C) Expression trends of specific genes along pseudotime. Splines were fitted using the monocle R package function. (D) Overlap of mast cell (MC) differentiation up- (i) or down- (ii) regulated genes with mast cell and basophil signature gene sets from Dwyer et al. Significance of overlap was tested using a hypergeometric test, with resulting p-values displayed in figure. The Venn diagrams show genes annotated in Ensembl genome build 81 and Dwyer et al.

Figure S3

(A) Flow cytometry assessment of colonies derived from single P1 peritoneal cells. The same colonies were stained with May-Grünwald Giemsa. (B) Cultured bulk-sorted peritoneal mast cells provided as reference to panel A. (C) Single-cells were sorted into individual wells and the colony size was determined after 7 days in culture. The numbers above each group represent the number of wells analyzed. Each dot represents one well. The red lines represent geometric means. The data in panel C are pooled from 2 independent experiments. Two-tailed Mann-Whitney test; ****P<0.0001. The cells were cultured with IL-3 and stem cell factor.

Figure S4

Flow cytometry plots of the FACS index data linked with the cell culture data (related to Figure 4F). (A) The colors represent $\log_{10}$-transformed colony size. (B) The colors represent colony type. The data is pooled from 2 independent experiments. Integrin β7 and side scatter (SSC) are visualized on log-transformed and linear scales, respectively.

Figure S5

(A) Flow cytometry gating strategy for determining colony type following culture for 6 days in erythrocyte-promoting conditions. E, erythroid; MC, mast cell; Ba, basophil. Culture of bulk-sorted bone marrow (BM) Lin$^-$ c-Kit$^+$ cells served as positive control for erythroid-forming potential. (B) Colony type output of single index-sorted P1 cells projected into the principal component space of the reference dataset. The point size represents $\log_{10}$-transformed colony size. (C) Single-cells were sorted into individual wells and the colony size was determined after 6 days in culture. The number represents the number of wells analyzed. Each dot represents one well. The red line represents

geometric mean. The data in panels B and C are pooled from 2 independent experiments.

Figure S6

(A) UMAP visualization of the Human Cell Atlas bone marrow data colored by expression of different genes (*CD34*, hematopoietic stem and progenitor cell signature; *MS4A2* and *HDC*, basophil-mast cell signature). (B) UMAP visualization of the HSPC subset, colored by cluster or expression of different genes (*MS4A2* and *HDC*, basophil-mast cell signature; *KLF1*, erythrocyte signature) (C) UMAP visualization of the basophil-mast cell subset colored by expression of different genes (*MS4A2*, basophil-mast cell signature; *CEBPA*, basophil signature; *TPSAB1*, mast cell signature).

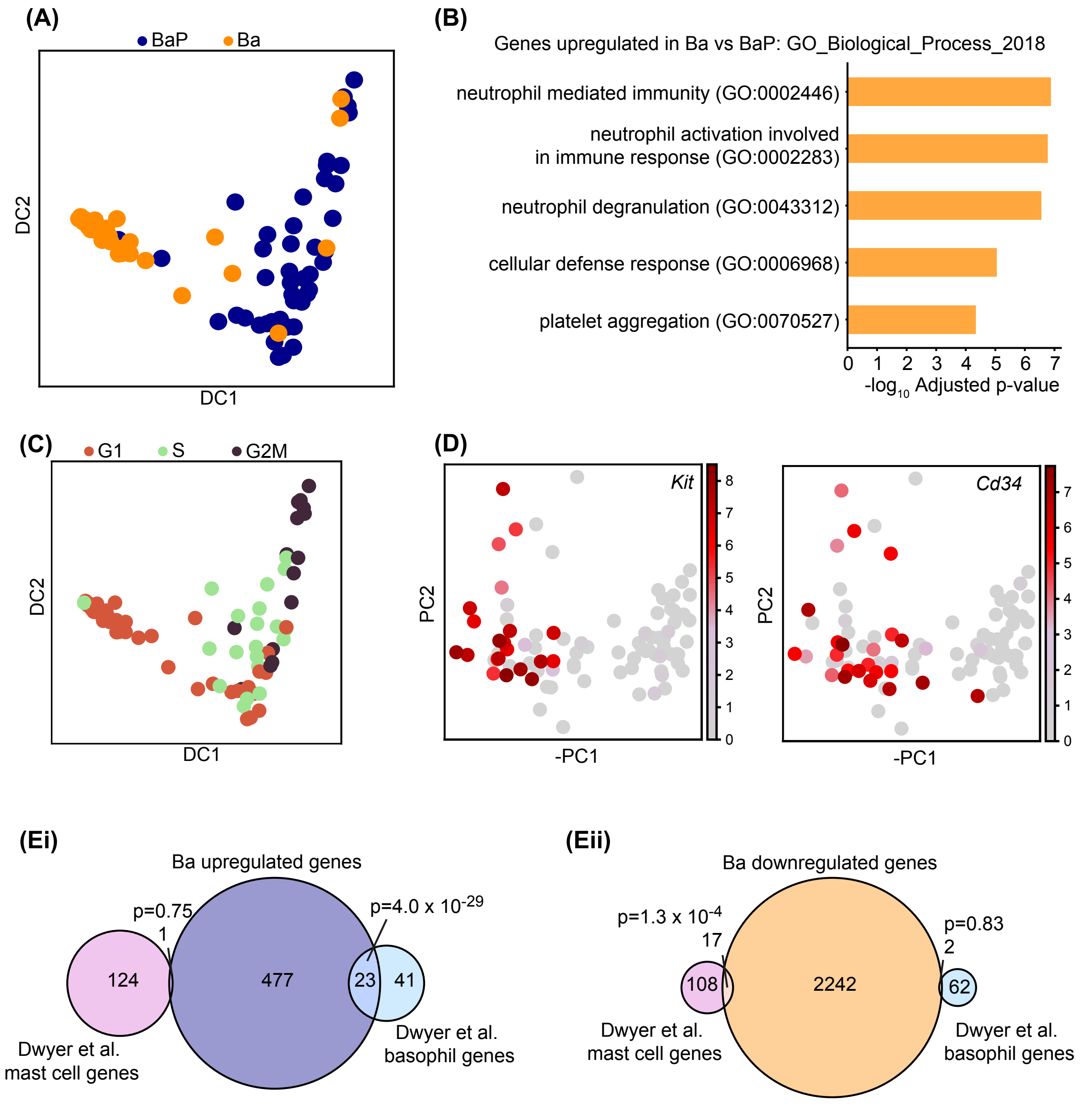

(A)
BaP
Ba
DC2
DC1
(B)
Genes upregulated in Ba vs BaP: GO_Biological_Process_2018
neutrophil mediated immunity (GO:0002446)
neutrophil activation involved in immune response (GO:0002283)
neutrophil degranulation (GO:0043312)
cellular defense response (GO:0006968)
platelet aggregation (GO:0070527)
0 1 2 3 4 5 6 7
-log10 Adjusted p-value
(C)
G1
S
G2M
DC2
DC1
(D)
Kit
Cd34
PC2
-PC1
(Ei)
Ba upregulated genes
p=0.75
1
124
477
p=4.0 x 10-29
23
41
Dwyer et al. mast cell genes
Dwyer et al. basophil genes
(Eii)
Ba downregulated genes
p=1.3 x 10-4
17
108
2242
p=0.83
2
62
Dwyer et al. mast cell genes
Dwyer et al. basophil genes

Figure S1

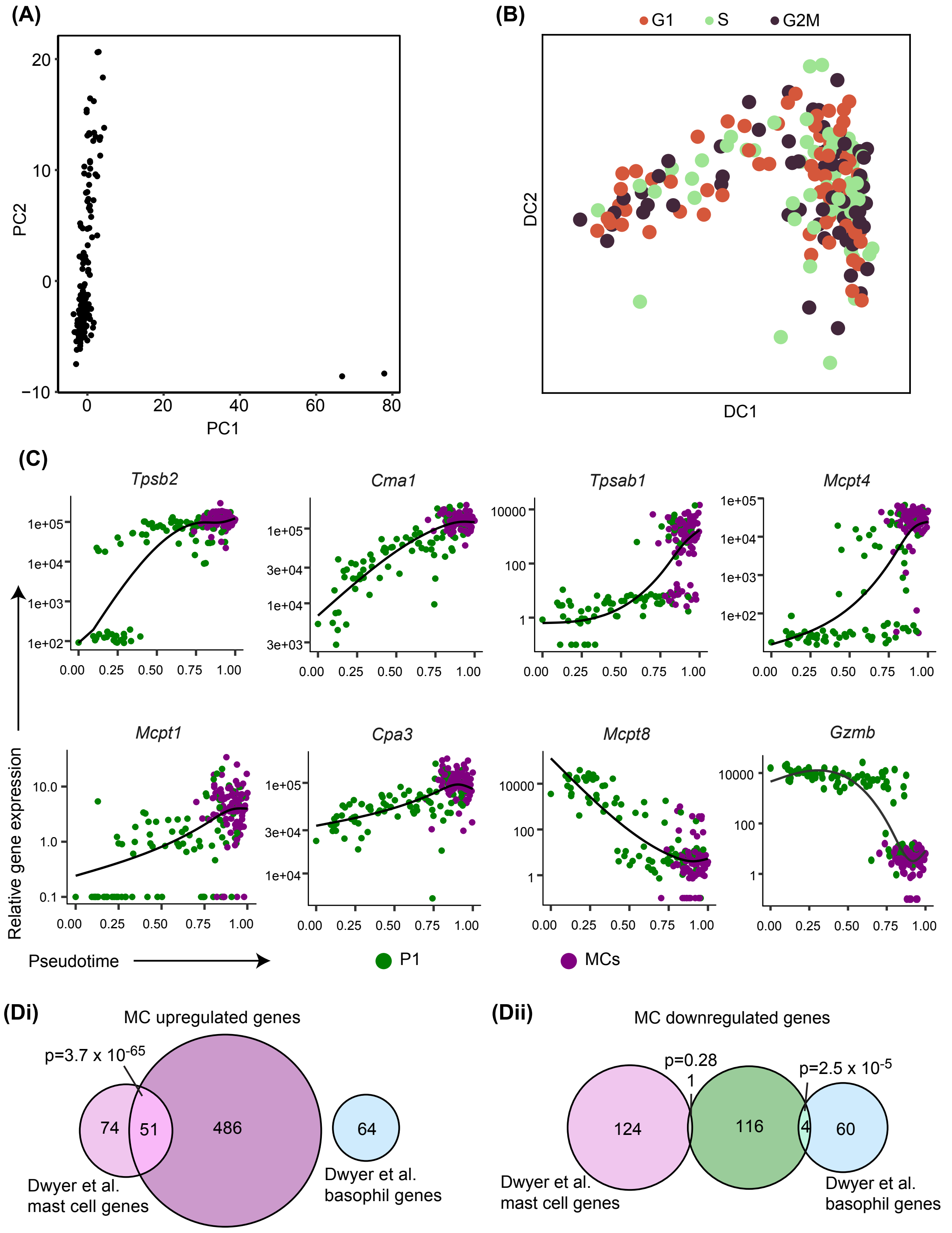

(A)
PC2
PC1
(B)
G1
S
G2M
DC2
DC1
(C)
Tpsb2
Cma1
Tpsab1
Mcpt4
Mcpt1
Cpa3
Mcpt8
Gzmb
Relative gene expression
Pseudotime
P1
MCs
(Di)
MC upregulated genes
p=3.7 x 10-65
74
51
486
64
Dwyer et al. mast cell genes
Dwyer et al. basophil genes
(Dii)
MC downregulated genes
p=0.28
1
p=2.5 x 10-5
124
116
4
60
Dwyer et al. mast cell genes
Dwyer et al. basophil genes

Figure S2

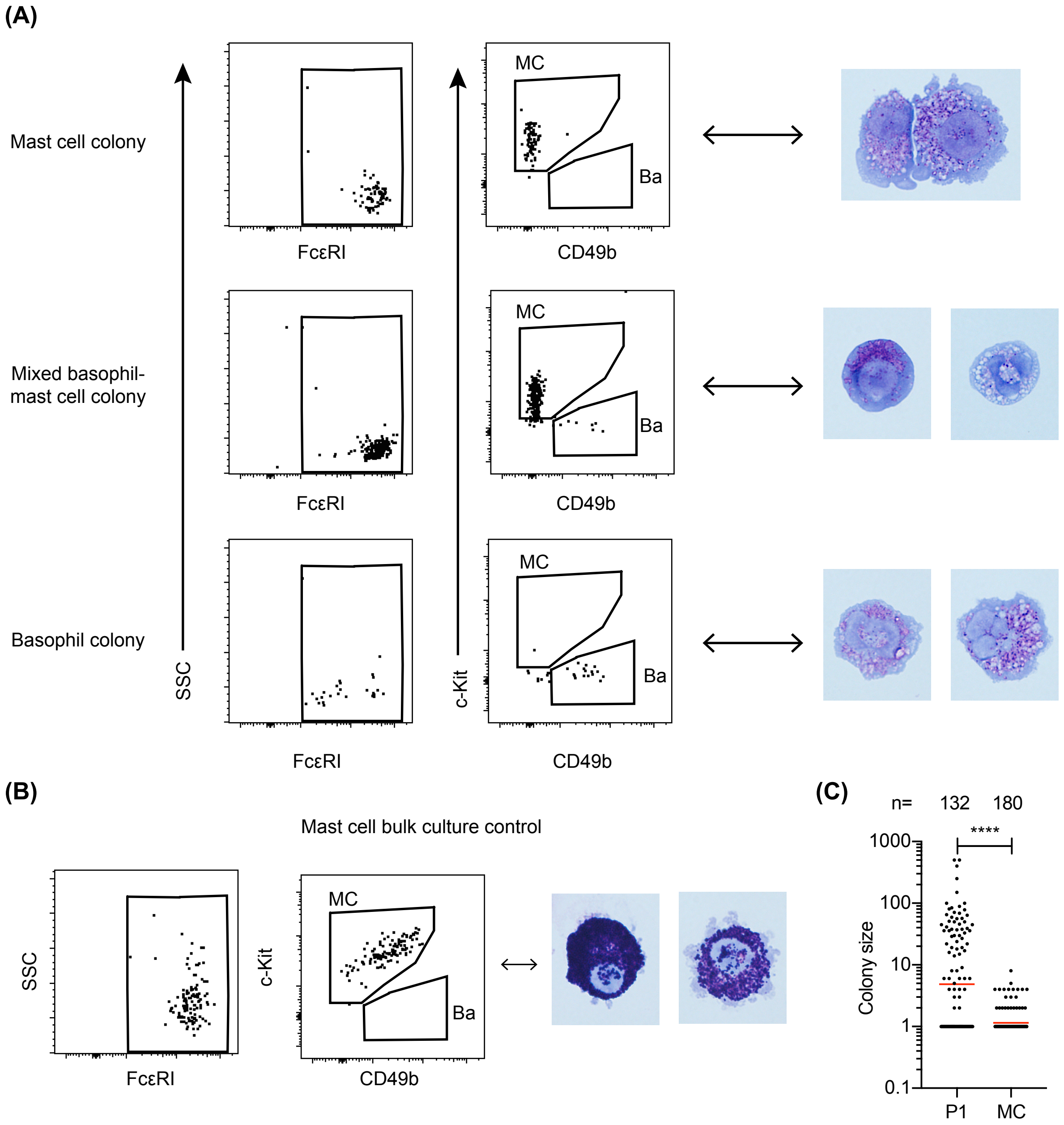

(A)
Mast cell colony
Mixed basophil-mast cell colony
Basophil colony
SSC
FcεRI
c-Kit
CD49b
MC
Ba
(B)
Mast cell bulk culture control
SSC
FcεRI
c-Kit
CD49b
MC
Ba
(C)
n=
132
180
****
1000
100
10
1
0.1
Colony size
P1
MC

Figure S3

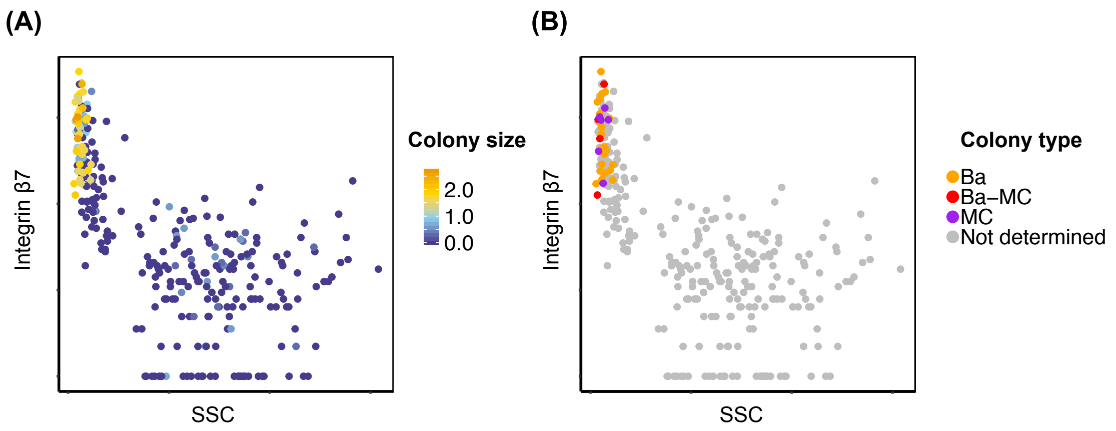
(A)
Integrin β7
SSC
Colony size
2.0
1.0
0.0
(B)
Integrin β7
SSC
Colony type
Ba
Ba–MC
MC
Not determined

Figure S4

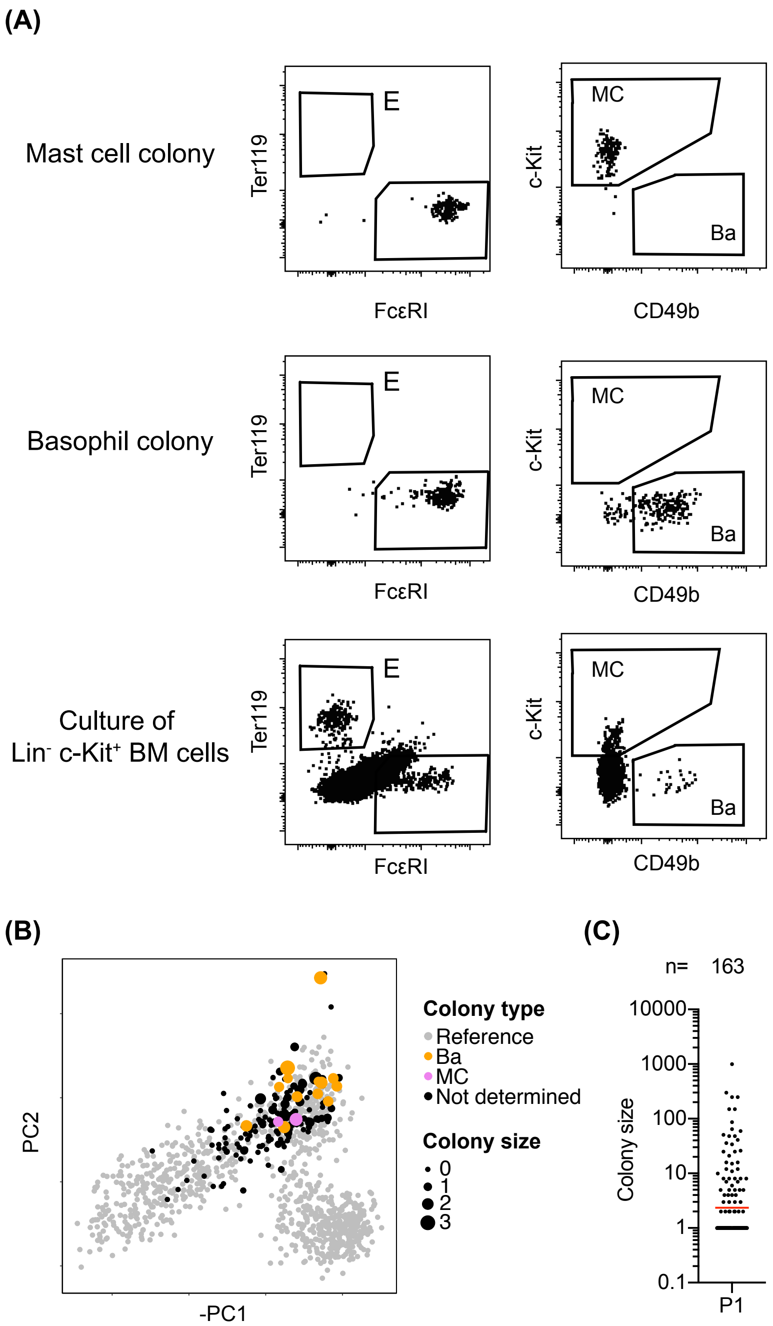
(A)
Mast cell colony
Basophil colony
Culture of
Lin- c-Kit+ BM cells
Ter119
FcεRI
E
c-Kit
CD49b
MC
Ba
(B)
PC2
-PC1
Colony type
Reference
Ba
MC
Not determined
Colony size
0
1
2
3
(C)
n= 163
Colony size
10000
1000
100
10
1
0.1
P1

Figure S5

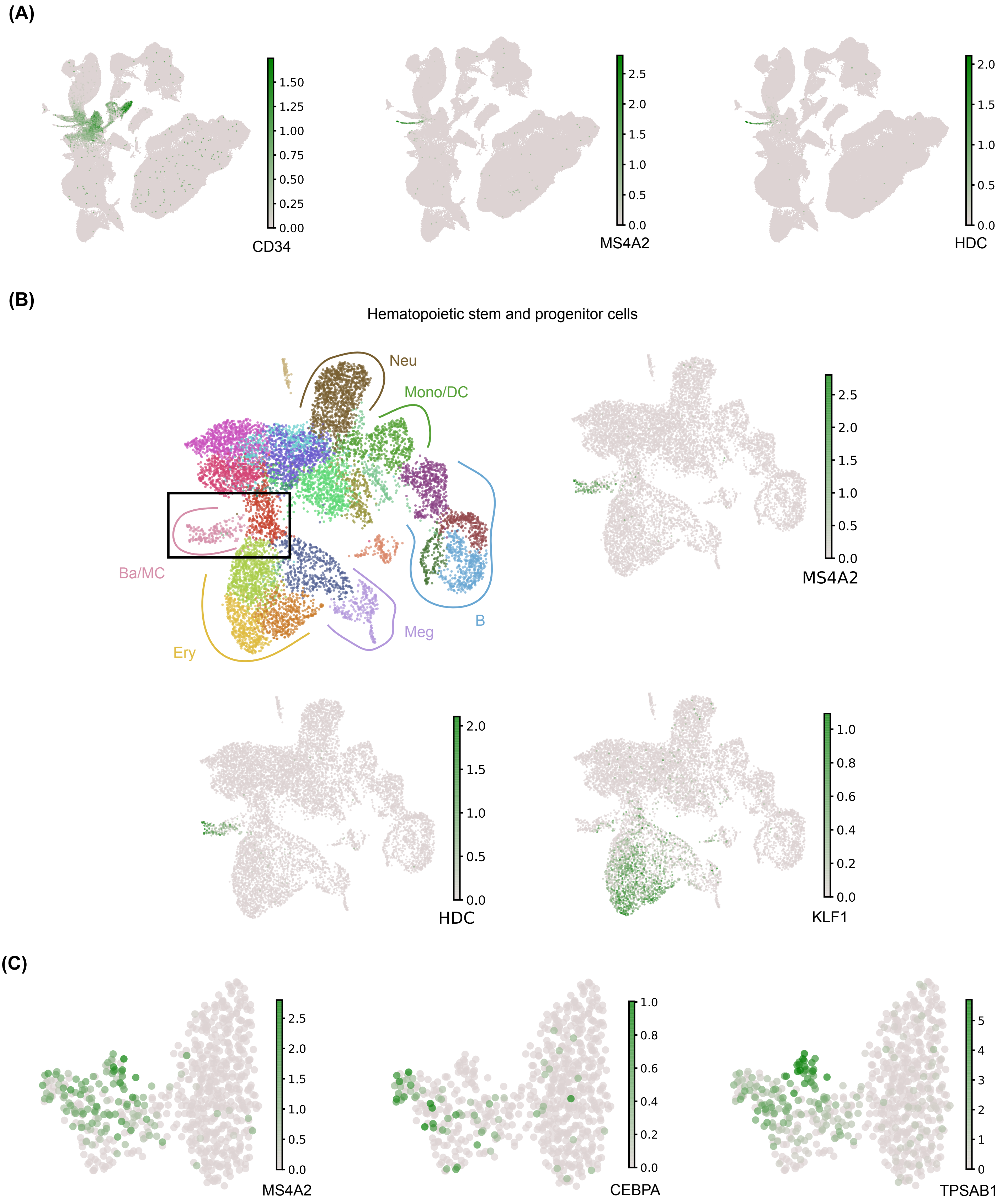
(A)
CD34
MS4A2
HDC
(B)
Hematopoietic stem and progenitor cells
Neu
Mono/DC
Ba/MC
B
Meg
Ery
MS4A2
HDC
KLF1
(C)
MS4A2
CEBPA
TPSAB1

Figure S6
