# Supplementary material for: Single‐cell molecular profiling provides a high‐resolution map of basophil and mast cell development
Source: Allergy. 2020 Nov 5;76(6):1731–42. doi: 10.1111/all.14633 (PMC8246912; doi:10.1111/all.14633)
Supplement: Supplementary file 6 — Supplementary Material [file ALL-76-1731-s002.docx]

**Supplementary methods**

*Preprocessing of mouse scRNA-seq data*

Mouse sequencing data were aligned using GSNAP^1^ to Ensembl genome build 81^2^ and gene counts were obtained using HT-Seq^3^. Quality control filtering and normalization was performed in the R programming environment. Quality control was performed to exclude cells with fewer than 500,000 reads mapping to nuclear genes or with over 25% of mapped reads mapping to ERCC spike-ins. For the peritoneal cells, principal component analysis (PCA) of the quality control-filtered samples showed that two cells separated in principal component (PC) 1 (Figure S2A). Genes with high PC1 loadings were highly significantly enriched for B cell related genes, so these two cells were suspected to be contaminating B cells and excluded from further analysis. Cells were then normalized using Scran^4^ and highly variable genes were identified using the ERCC spike-ins to estimate technical variance.^5^ This identified 3330 highly variable genes for the basophil dataset and 1832 highly variable genes for the mast cell dataset.

*Data visualization and pseudotime ordering*

Inbuilt scanpy functions were used for PCA and diffusion map dimensionality reduction. Differential expression between cell types was performed using the *rank_genes_groups* function with the t-test_overestim_var method. P-values were adjusted using the benjamini-hochberg procedure, and genes with adjusted p-value < 0.01 were considered significant. Gene list enrichment analysis was performed using the *enrichr* function from the *gseapy* python module.^6,7^ Cell cycle scoring was performed on scaled data using the scanpy *score_genes_cell_cycle* function with S and G2/M phase gene lists from Macosko et al^8^. Peritoneal mast cells were ordered in pseudotime using the diffusion pseudotime (DPT) scanpy implementation.^9^ Due to cell cycle effects confounding the diffusion map and DPT analysis, basophil progenitor cells were ordered in pseudotime by ordering cells along PC1. Plotting computationally assigned cell cycle states in the peritoneal mast cell diffusion map showed that confounding cell cycle effects were not present in these data (Figure S2B).

*Identification of dynamically regulated pseudotime genes*

Genes with dynamic expression in pseudotime were identified following the method of Tusi et al^10^. Gene expression was first smoothed along pseudotime using a sliding window of size 20. For each ordering, the windows with minimum and maximum gene expression were identified, and a t-test performed between the values in each of these windows, giving a p-value for each gene. To generate a background distribution, this analysis was repeated for a random shuffling of cells along pseudotime. The adjusted p-value for each gene was then calculated as the fraction of shuffled p-values across all genes that were less than the p-value of the gene in question for non-permuted data. Genes with adjusted p-value < 0.01 were then treated as dynamic across pseudotime and plotted in the heatmaps. Gene expression in the heatmap was smoothed using a sliding window of size 20 and z-score transformed for each gene. To identify upregulated and downregulated gene groups, genes were clustered using Louvain clustering^11^ implemented in scanpy with resolution=2. Mast cell and basophil signature gene sets were obtained from Dwyer et al.^12^ Mast cell signature genes were those found upregulated in murine mast cell populations (taken from trachea, tongue, esophagus, skin and peritoneum) compared to several other immune cell types profiled using microarrays and were obtained from Figure 3a in Dwyer et al. Basophil signature genes were those found by Dwyer et al to be upregulated in basophils from blood and spleen compared to several other immune cell types and were obtained from Figure 4a in the original publication. Statistical overlap between gene lists was calculated using a hypergeometric test. Panther v14.1 was used to identify the dynamic mast cell genes annotated as proteases (Panther category PC00190)^13^. To plot gene expression trends along pseudotime the genSmooth Curves function from the monocle R package^14^ was used to fit smooth spline curves for the expression of each gene against pseudotime. When all genes were plotted together expression values of each gene were scaled by dividing values by the maximum of that gene to account for the very different dynamic ranges across genes.

*Processing of Human Cell Atlas data*

Quality control was performed to exclude cells with less than 600 genes detected, or with >10% UMI counts mapped to mitochondrial genes. Data was analyzed using the scanpy package using the following steps: expression normalization by the total counts per cell, log-transformation, scaling, identification of highly variable genes, calculation of principal components, identification of nearest neighbors, clustering using the Leiden algorithm and dimensionality reduction using the UMAP algorithm.^15^ The whole dataset of bone marrow mononuclear cells was first subset for clusters corresponding to HSPCs and subsequently for cells connected to the basophil and mast cell trajectories (Figure 4G-H, S6B). At each step cell counts were re-processed, re-clustered and new UMAP coordinates were calculated. Identity of the clusters was assigned based on established marker genes, as indicated in the figures. Connections between clusters were verified using partition-based graph abstraction^16^ and diffusion map methods.

**References**

1. Wu TD, Nacu S. Fast and SNP-tolerant detection of complex variants and splicing in short reads. *Bioinformatics.* 2010;26:873-881.

2. Zerbino DR, Achuthan P, Akanni W, et al. Ensembl 2018. *Nucleic Acids Res.* 2018;46:D754-D761.

3. Anders S, Pyl PT, Huber W. HTSeq--a Python framework to work with high-throughput sequencing data. *Bioinformatics.* 2015;31:166-169.

4. Lun AT, Bach K, Marioni JC. Pooling across cells to normalize single-cell RNA sequencing data with many zero counts. *Genome Biol.* 2016;17:75.

5. Brennecke P, Anders S, Kim JK, et al. Accounting for technical noise in single-cell RNA-seq experiments. *Nat Methods.* 2013;10:1093-1095.

6. Chen EY, Tan CM, Kou Y, et al. Enrichr: interactive and collaborative HTML5 gene list enrichment analysis tool. *BMC Bioinformatics.* 2013;14:128.

7. Kuleshov MV, Jones MR, Rouillard AD, et al. Enrichr: a comprehensive gene set enrichment analysis web server 2016 update. *Nucleic Acids Res.* 2016;44:W90-97.

8. Macosko EZ, Basu A, Satija R, et al. Highly Parallel Genome-wide Expression Profiling of Individual Cells Using Nanoliter Droplets. *Cell.* 2015;161:1202-1214.

9. Haghverdi L, Buttner M, Wolf FA, Buettner F, Theis FJ. Diffusion pseudotime robustly reconstructs lineage branching. *Nat Methods.* 2016;13:845-848.

10. Tusi BK, Wolock SL, Weinreb C, et al. Population snapshots predict early haematopoietic and erythroid hierarchies. *Nature.* 2018;555:54-60.

11. Blondel VD, Guillaume J-L, Lambiotte R, Lefebvre E. Fast unfolding of communities in large networks. *Journal of Statistical Mechanics: Theory and Experiment.* 2008;2008.

12. Dwyer DF, Barrett NA, Austen KF, Immunological Genome Project C. Expression profiling of constitutive mast cells reveals a unique identity within the immune system. *Nat Immunol.* 2016;17:878-887.

13. Mi H, Muruganujan A, Ebert D, Huang X, Thomas PD. PANTHER version 14: more genomes, a new PANTHER GO-slim and improvements in enrichment analysis tools. *Nucleic Acids Res.* 2019;47:D419-D426.

14. Trapnell C, Cacchiarelli D, Grimsby J, et al. The dynamics and regulators of cell fate decisions are revealed by pseudotemporal ordering of single cells. *Nat Biotechnol.* 2014;32:381-386.

15. Kucinski I, Wilson NK, Hannah R, et al. Interactions between lineage-associated transcription factors govern haematopoietic progenitor states. *EMBO J.* 2020:e104983.

16. Wolf FA, Hamey FK, Plass M, et al. PAGA: graph abstraction reconciles clustering with trajectory inference through a topology preserving map of single cells. *Genome Biol.* 2019;20:59.
